# Viral Burden of Respiratory Syncytial Virus and Viral Coinfections as Factors Regulating Paediatric Disease Severity

**DOI:** 10.3390/v17091236

**Published:** 2025-09-11

**Authors:** Velia Chiara Di Maio, Rossana Scutari, Martina Mastropaolo, Luna Colagrossi, Giulia Linardos, Luana Coltella, Stefania Ranno, Eugenia Galeno, Mara Pisani, Anna Chiara Vittucci, Sebastian Cristaldi, Massimiliano Raponi, Alberto Villani, Cristina Russo, Carlo Federico Perno

**Affiliations:** 1 Microbiology and Diagnostic Immunology Unit, Bambino Gesù Children’s Hospital, IRCSS, 00165 Rome, Italy; veliachiara.dimaio@opbg.net (V.C.D.M.); carlofederico.perno@opbg.net (C.F.P.); 2 Multimodal Laboratory Research Unit, Bambino Gesù Children’s Hospital, IRCCS, 00165 Rome, Italy; 3 Paediatrics Clinical Unit, Bambino Gesù Children’s Hospital, IRCCS, 00165 Rome, Italy; 4 Medical Direction, Bambino Gesù Children’s Hospital, IRCCS, 00165 Rome, Italy

**Keywords:** respiratory syncytial virus, disease severity, paediatric patients, co-infection, disease burden

## Abstract

Background: Respiratory syncytial virus (RSV) is a leading cause of acute respiratory infections (ARIs) in children. However, the clinical impact of RSV co-infection with other respiratory viruses remains unclear. This study investigates the frequency and clinical outcomes of RSV infections in a large paediatric cohort. Methods: Paediatric patients with RSV-positive respiratory samples admitted to Bambino Gesù Children’s Hospital between January 2022 and April 2024 were analysed. Results: Within 17,259 respiratory samples from 9877 paediatric patients, 952 (9.6%) were RSV-positive. Among these, 637 patients with ARI were included. RSV affected the lower respiratory tract in 549 cases (86.2%) and the upper tract in 88 (13.8%) cases. RSV mono-infection was found in 286 (44.9%) patients, while 351 (55.1%) patients had co-infections. Mono-infections showed lower cycle-threshold (CT) than co-infections in both upper and lower tract (*p*-value:0.002 and 0.037, respectively). Pneumonia was associated with RSV co-infection (N = 48, 15.4%), whereas bronchiolitis was mostly seen in mono-infection (N = 196, 78.1%, *p*-value:0.002). Regression analysis showed an association between pneumonia and co-infection (AOR: 1.97 [1.06–3.64], *p*-value = 0.031), higher CT (AOR [95% CI]: 1.07 [1.02–1.11], *p*-value = 0.006) and older age (AOR [95% CI]: 1.48 [1.31–1.68], *p*-value < 0.001), whereas bronchiolitis was associated with mono-infection, younger age and lower CT. Conclusions: This study highlights the role of RSV in paediatric disease and emphasises the importance of early diagnosis, personalised treatment and preventive strategies to improve outcomes and reduce the burden of disease.

## 1. Introduction

Respiratory syncytial virus (RSV) is the leading cause of acute respiratory infection (ARI) during childhood, causing significant morbidity and mortality, especially in infants and in the early years of life [1]. Moreover, RSV is also an important pathogen causing severe infections in elderly and immunocompromised patients [2]. RSV is an enveloped virus with a non-segmented negative-sense RNA genome of approximately 15,200 nucleotides, belonging to the Pneumovirus family, of the genus Orthopneumovirus [3]. Based on its antigenic and genetic variability, RSV is classified into two subtypes: A and B (RSV-A and RSV-B). In areas with a temperate climate, RSV infection exhibits a seasonal epidemic that typically occurs annually, peaking in the winter months.

RSV infection has a wide range of clinical presentations, ranging from minor upper respiratory tract infection (URTI) or otitis media to severe lower respiratory tract infections (LTRIs), like bronchiolitis and pneumonia. RSV infection is a major cause of severe LRTI in young infants, including those who are otherwise healthy babies without pre-existing medical conditions [4]. Severe paediatric RSV disease in young infants may require hospitalisation and intensive care unit (ICU)-level care and can be fatal. In addition to acute disease, RSV infection can lead to long-term respiratory sequelae such as recurrent wheezing and asthma, particularly in children with severe bronchiolitis [5]. Several risk factors could influence the development of severe RSV infection in paediatric patients, including preterm birth, younger age at the time of infection, and viral co-infection [6].

Before 2023, palivizumab was the only monoclonal antibody available as a prophylactic for protecting infants against RSV [4]. However, the use of this monoclonal antibody is still largely limited to high-risk infants due to its high cost and relatively short half-life, which requires frequent administrations (Synagis [palivizumab] prescribing information).

Recently, two effective strategies for infant protection have been introduced: a maternal vaccine (Abrysvo) and a long-acting monoclonal antibody (Nirsevimab) [4].

Abrysvo is a protein subunit vaccine that consists of a recombinant RSV F protein antigen, based on both RSV-A and RSV-B subtypes, and stabilised in the prefusion (preF) conformation. It is administered during weeks 32–36 of pregnancy, enabling the transfer of protective antibodies to the infant before birth. Clinical trials have shown that the vaccine provides significant efficacy in preventing severe RSV disease in infants during the first 3 to 6 months of life, demonstrating 81.8% efficacy within 90 days of birth and 69.4% efficacy within 180 days [7,8].

Nirsevimab is a long-acting monoclonal antibody that provides passive protection against RSV in infants by targeting a conserved epitope on the F1 and F2 subunits of the RSV fusion protein, locking it in its prefusion form to block viral entry. Nirsevimab offers at least 5 months of protection with a single dose, effectively covering a full RSV season. It has shown up to 90% efficacy in preventing RSV-related illness and reduces the need for repeated dosing compared to palivizumab [9,10].

Both RSV vaccines and long-acting monoclonal antibodies administered to infants have the potential to significantly reduce the disease burden and severe outcomes of RSV in young infants [11]. However, the effectiveness of these new and promising preventive strategies still requires extensive confirmation in clinical practice. In particular, it remains unclear in which patients and under which conditions RSV is associated with a worse prognosis, and whether coinfections with concurrent viruses may affect the clinical outcome.

In this context, the study aims to analyse the frequency of RSV infections and related clinical outcomes in about 1000 children followed at a large Italian tertiary paediatric hospital. Specifically, the primary objectives are (1) to determine the prevalence of RSV mono-infections and co-infections with other respiratory viruses and (2) to assess the clinical impact of these infectious profiles in terms of disease severity, type of respiratory involvement (upper vs. lower tract) and associated clinical diagnoses (e.g., bronchiolitis, pneumonia).

These objectives aim to provide a more in-depth understanding of the overall impact of RSV infections on the pathogenesis and clinical management of paediatric respiratory infections, with possible implications for improving diagnostic and therapeutic strategies.

## 2. Materials and Methods

### 2.1. Sample Characteristics and Inclusion Criteria of the Analysis

This retrospective observational study included RSV-positive respiratory samples, obtained from children and adolescents (0–18 years) referred to Bambino Gesù Children Hospital IRCCS between 1 January 2022 and 30 April 2024. The RSV-positive samples were selected according to the criteria defined in Supplementary Figure S1.

The study included children and adolescents aged 0–18 years who tested positive for RSV and had a confirmed diagnosis of ARI at the time of sampling, as well as a SARS-CoV-2 test performed within 72 h before or after the RSV test. Patients who tested positive for RSV but did not have a documented ARI diagnosis were excluded from the study, as were those for whom the SARS-CoV-2 test was not performed within the specified timeframe. Additionally, samples without sufficient clinical data to accurately classify upper or lower respiratory tract involvement were excluded.

For all included patients, clinical data related to the diagnosis of ARI (either upper or lower respiratory tract infections) were collected and analysed. Upper ARI and Lower ARI were defined according to the guidelines from *Respiratory Tract Infections—Antibiotic Prescribing: Prescribing of Antibiotics for Self-Limiting Respiratory Tract Infections in Adults and Children in Primary Care* [12] and based on clinical features and chest radiograph imaging. Specifically, upper ARI includes clinical conditions such as the common cold, laryngitis, pharyngitis/tonsillitis, acute rhinitis, rhinosinusitis, and otitis media. Lower ARI encompasses conditions such as bronchiolitis, pneumonia, and asthmatic bronchitis.

The final assessment of both upper and lower ARI was performed at hospital admission and at the time of respiratory sample collection.

The study population was also stratified by age group according to the terminology established by the Eunice Kennedy Shriver National Institute of Child Health and Human Development [13,14].

### 2.2. Ethics Committee Statement

This study was conducted in accordance with the principles of the 1964 Declaration of Helsinki. In accordance with hospital regulations regarding retrospective observational studies, informed consent was waived.

As the study was non-interventional, observational and retrospective, involving only the analysis of anonymised routine clinical data, and not including any experimental procedures or direct patient interaction, approval by the Ethics Committee was not required. This approach is consistent with the standards established for non-invasive studies based on routine clinical data. All data were anonymised to ensure the protection of patient privacy and confidentiality.

### 2.3. Multiplex Respiratory Virus Panel

Respiratory viruses were assessed using the Allplex Respiratory Panel assay (Seegene Inc., Seoul, Republic of Korea), a multiplex one-step real-time RT-PCR system for the simultaneous detection of several respiratory viral pathogens.

This closed multiplex PCR system includes extraction, amplification, detection, and analysis of samples, with all steps carried out automatically.

The respiratory samples were routine clinical specimens collected using nasopharyngeal swabs in Universal Transport Medium (UTM).

Samples were processed promptly after collection, and if immediate processing was not possible, the samples were stored at −20 °C for no more than a few days.

Nucleic acid extraction (RNA and DNA) and PCR preparation were carried out in a single step using a Hamilton system (Seegene Inc., Seoul, Republic of Korea) based on the magnetic bead method, while multiplex RT-PCR amplification was performed using a CFX96™ Real-time PCR System (Bio-Rad Laboratories, Hercules, CA, USA).

The assay is able to identify 16 different respiratory viruses by using three different panels. These panels allow the detection of: Adenovirus (AdV); Human-Bocavirus (HBoV); Human-Coronavirus 229E, NL63 and OC43 (HCoV 229E/NL63/OC43); Human-Enterovirus (HeV); Influenza-A and -B (Flu-A and -B); Human-Metapneumovirus (HMPV); Parainfluenza Viruses (PIV)-1-4; Human-Rhinovirus (HRV); Respiratory Syncytial Virus-A and -B (RSV-A and -B).

The results were interpreted according to the manufacturer’s guidelines and automatically analysed using Seegene Viewer V3.0. The assay provides cycle threshold (Ct) values for all positive targets. In accordance with the datasheet specifications, a sample was considered positive if the Ct value was ≤42. A sample was considered negative when no amplification of any target pathogen was detected, provided that the internal control was successfully amplified.

### 2.4. Severe Acute Respiratory Syndrome-CoronaVirus 2 (SARS-CoV-2) Detection

SARS-CoV-2 results assessed by antigenic or molecular testing were integrated with multiplex respiratory panel data.

SARS-CoV-2 antigen testing was conducted using either the Roche Elecsys SARS-CoV-2 Antigen (Roche Diagnostics GmbH, Mannheim, Germany) or the fluorescent immunoassay RADT STANDARD F COVID-19 Ag FIA (SD BIOSENSOR, Suwon, Republic of Korea).

The Roche Elecsys SARS-CoV-2 Antigen assay utilises a sandwich immunoassay technique to detect viral nucleocapsid protein. The RADT STANDARD F COVID-19 Ag FIA is a rapid antigen test employing lateral flow technology to detect the SARS-CoV-2 spike protein antigen in respiratory samples.

A result with a cut-off index (COI) ≥ 1.0 was interpreted as reactive for SARS-CoV-2 antigen according to the manufacturer’s instructions.

Molecular tests were performed using the Cepheid Xpert Xpress CoV-2 plus kit (Cepheid, Sunnyvale, CA, USA) or the SARS-CoV-2 ELITe MGB Kit^®^ kit (Elitechgroup, Turin, Italy). All tests were performed in accordance with the manufacturer’s instructions, and results were interpreted using the recommended cut-off values.

### 2.5. Interpretation of Data and Statistical Analysis

Based on the results obtained for other microorganisms, samples were classified as positive only for RSV or positive for RSV and other viruses. Co-infection was defined as positive detection of RSV and at least one other virus in the same sample.

Descriptive statistics are presented as median values with interquartile range (IQR) for continuous variables and numbers (percentages) for categorical variables. Mann–Whitney and Fisher’s exact test were used to estimate potential associations between demographic and clinical characteristics and the type of infection. Univariate and multivariate logistic regression analyses were conducted to identify demographic, virus-related, and clinical factors independently associated with pneumonia. All variables from the univariate analysis were included in the multivariate logistic regression model, and a forward conditional stepwise selection method was applied to identify the factors independently associated with pneumonia.

The Spearman’s rank correlation coefficient was used to estimate correlations between RSV co-infection and other respiratory viruses. Two-sided *p*-values were always reported. A *p*-value < 0.05 was considered statistically significant. Statistical analyses were performed with SPSS software package (v29.0; SPSS Inc., Chicago, IL, USA).

## 3. Results

### 3.1. Study Population and Sampling Criteria

Between 1 January 2022, and 30 April 2024, a total of 17,259 respiratory samples were collected from 9877 children and adolescents aged 0 to 18 years with clinically relevant respiratory symptoms and analysed using the Allplex Respiratory Panel Assays at the Bambino Gesù Children’s Hospital in Rome (Supplementary Figure S1). Among these patients, 952 (9.6%) tested positive for RSV, confirming the persistent and clinically relevant role of RSV as one of the main causative agents of respiratory infections in children.

Among the RSV-positive cases, 690 patients had also undergone an antigenic or molecular test for SARS-CoV-2 detection performed within 72 h before or after Allplex Respiratory Panel Assays, allowing for an accurate assessment of the frequency of viral co-infections and any overlap in epidemic dynamics. Of these, 637 patients had a documented diagnosis of acute respiratory infection at the time of sampling and were included in the final study population.

The final study population therefore consisted of 637 children and adolescents who tested positive for RSV, were diagnosed with ARI and had SARS-CoV-2 tests available. These patients were then stratified according to the anatomical site of respiratory involvement. Specifically, 88 patients were classified as having upper respiratory tract infections, while the majority (549 patients) had lower respiratory tract infections, reflecting the well-known propensity of RSV to cause severe lower airway involvement. The inclusion criteria and patient stratification are illustrated in Supplementary Figure S1.

### 3.2. Seasonal Distribution of RSV During Study Period

Looking at the seasonal pattern of RSV during the study period, as expected, RSV in the paediatric population was detected mainly during the winter months in all years of the study [15].

In the 2022–2023 season, RSV cases increased rapidly from November, reaching the highest peak in December 2022, and remaining elevated until February 2023. The 2023–2024 season showed a similar temporal pattern but with a slightly lower intensity, with the main peak again in December and a gradual decline in January–February 2024.

Considering RSV type (RSV-A and RSV-B), a different seasonal distribution between 2022 and the early part of 2024 was observed. In particular, during the cold months of November to December 2022 and January to February 2023, RSV-B was more common than RSV-A (85.5% RSV-B and 14.5% RSV-A). In contrast, between November 2023 and February 2024, RSV-A predominated over RSV-B (76.2% RSV-A and 23.8% RSV-B) (Supplementary Figure S2).

### 3.3. Patients’ Characteristics

Of the 637 RSV-positive patients with ARI diagnosed, 331 (52.0%) were male. The median age was 4.7 (IQR: 1.8–20.9) months. Most of the subjects were less than 1 year old (neonates and infants; N = 413, 64.8%), followed by early childhood (2.1–5 years; N = 117, 18.4%), toddlers (13 months to 2 years; N = 75, 11.8%), middle childhood (6 to 11 years; N = 21, 3.3%), and adolescents (12 to 18 years; N = 11, 1.7%).

Nearly all respiratory specimens (99.8%) were collected by a nasopharyngeal aspirate (N = 486, 76.3%) or nasopharyngeal swab (N = 150, 23.5%; Table 1).

At the time of testing, the most common disease was bronchiolitis (N = 373, 58.6%), followed by asthmatic bronchitis (N = 108, 17.0%) and pneumonia (N = 68, 10.7%). Almost all patients were hospitalised (N = 631, 99.1%).

Most RSV infections (N = 549, 86.2%) involved children who had an ARI associated with the lower respiratory tract, and all remaining (N = 88, 13.8%) involved patients with upper respiratory infections (Table 1).

Looking at the differences in demographic and clinical characteristics between the two groups (upper and lower respiratory tract involvement), patients with lower respiratory infection were younger than those with upper respiratory tract infection (3.5 [1.7–16.2] vs. 31.1 [10.5–58.6] months, *p*-value < 0.001). In particular, neonates were more likely to have lower respiratory tract manifestations compared with upper infections (9.5% vs. 1.1%, *p*-value = 0.006). A similar observation was seen in infants (61.4% lower ARI vs. 26.1% upper ARI, *p*-value < 0.001). In contrast, early childhood and adolescence had more upper than lower tract infections (Upper: 42% and 5.7% vs. Lower: 14.6% and 1.1%, *p*-value < 0.001 early childhood and 0.011 adolescence).

No significant differences were observed between the two groups in terms of sample type and RSV genotype infections.

Demographic and clinical characteristics of patients according to ARI are reported in Table 1.

### 3.4. Detection of Respiratory Virus

**Figure 1:**
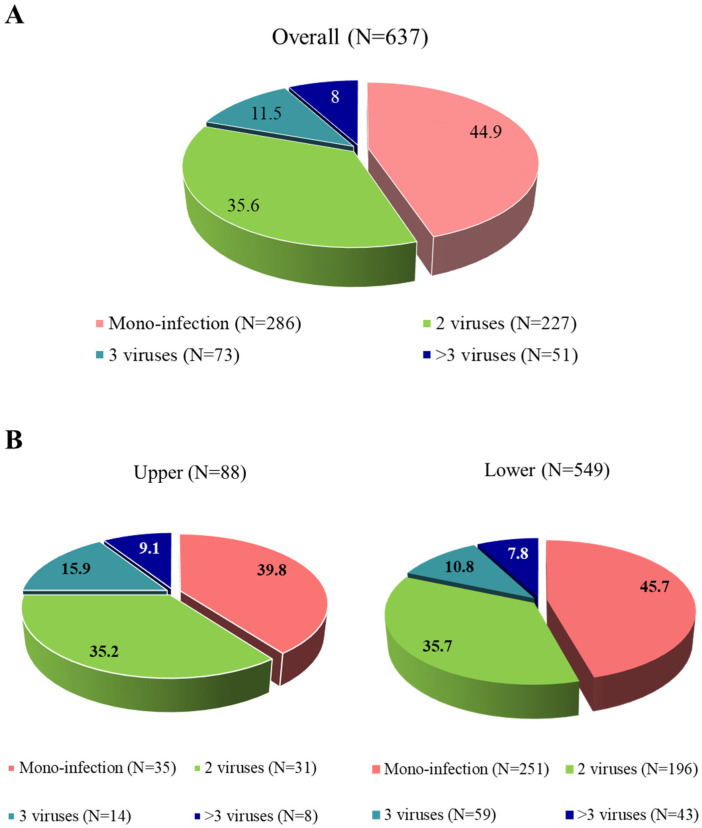
Distribution of viral detections in positive respiratory samples with a SARS-CoV-2 test available in the overall population (**A**) and according to upper and lower respiratory tract infections (**B**).

Analysis of RSV detection showed that 44.9% (N = 286) of patients had an RSV mono-infection, while 55.1% (N = 351) were positive for RSV together with at least one other virus. Co-infections often involved two additional viruses (35.6%, N = 227), while infections with more than three viruses were less frequent but still notable (11.5%, N = 73 for three viruses and 8.0%, N = 51 for more than three viruses; Figure 1A). When comparing viral co-infections in upper and lower ARIs, no statistical difference was found (60.2% vs. 54.3%, *p*-value = 0.356; Figure 1B).

Among the RSV co-infections, more than half involved HRV (N = 220, 62.7%), followed by HBoV (N = 70, 19.9%), HeV (N = 55, 15.7%), AdV (N = 54, 15.4%) and OC43 (N = 42, 12.0%) (the sum is greater than 100% because of triple and quadruple co-infections) (Supplementary Figure S3A). RSV co-infections showed a clear seasonal pattern, with a marked predominance in winter (January–March and October–December), while the summer months recorded lower frequencies, in line with the peak circulation of RSV (Supplementary Table S1).

These viruses were evenly distributed between upper and lower respiratory tract infections (including asthmatic bronchitis, bronchiolitis and pneumonia) when co-infected with RSV, with exceptions: SARS-CoV-2 co-infection was present in 6.3% individuals (N = 22), and more common with RSV in upper respiratory tract than in lower respiratory tract infections (20.8% vs. 3.7%, *p*-value < 0.001), while OC43 was more commonly observed in lower respiratory tract infections than in upper respiratory tract infections (13.4% vs. 3.8%, *p*-value = 0.063; Supplementary Figure S3B).

### 3.5. RSV Cycle Threshold Values (CT)

The median (IQR) RSV viral load, as measured by RSV CT, was 22.9 (19.4–27.9). When analysing the two RSV subtypes separately, slightly lower (though significant, *p*-value < 0.001) CT values were observed for RSV-B (median [IQR]: 22.3 [18.3–27.6]) compared to RSV-A (median [IQR]: 23.6 [20.5–28.1]).

**Figure 2:**
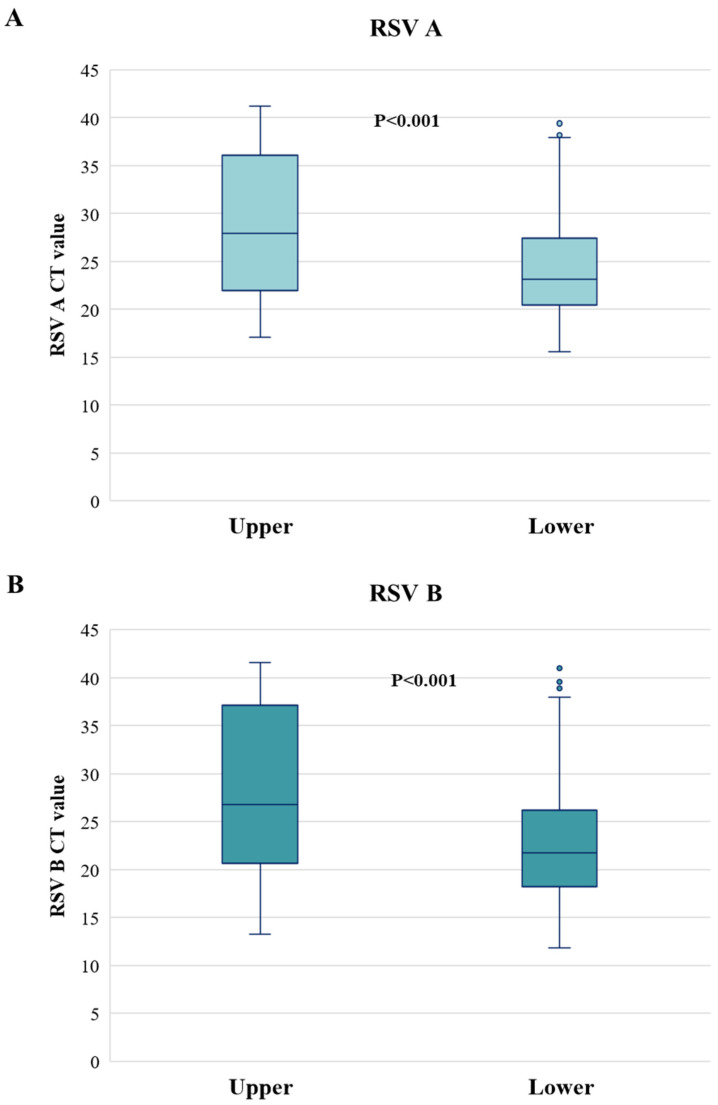
Cycle threshold values of RSV A (**A**) and RSV B (**B**) according to upper and lower respiratory tract infections. CT: cycle threshold. Two-sided *p*-value was calculated by Mann–Whitney test.

Looking at the RSV viral load in upper and lower respiratory tract infection, significantly higher CT values (corresponding to a lower viral-load) were found for both RSV-A and -B in upper respiratory tract (Median [IQR]; RSV-A: 28.0 [21.9–35.6], RSV-B: 26.8 [20.6–37.1]) than in lower respiratory tract infection (RSV-A: 23.1 [20.5–27.5], RSV-B: 21.7 [18.2–26.2]) (*p*-value < 0.001) (Figure 2).

This higher viral load in the lower respiratory tract infection occurs despite the tests being performed upon nasopharyngeal swabs that, by definition, capture mostly the virus present in the upper respiratory tract; this supports that the viral load present in the lower respiratory tract during RSV infection is remarkably higher than in the upper respiratory tract.

On the other hand, when comparing viral loads in mono- and co-infections, significantly lower CT levels were observed in mono-infections compared to RSV co-infections (Mono-infection: 25.2 [19.6–28.4] upper and 22.0 [18.9–25.7] lower; Co-infections: 32.1 [24.3–38.3] upper and 22.9 [19.7–27.4] lower; *p*-value = 0.002 for upper and 0.037 for lower) (Table 2).

### 3.6. Correlation with Pneumonia

**Figure 3:**
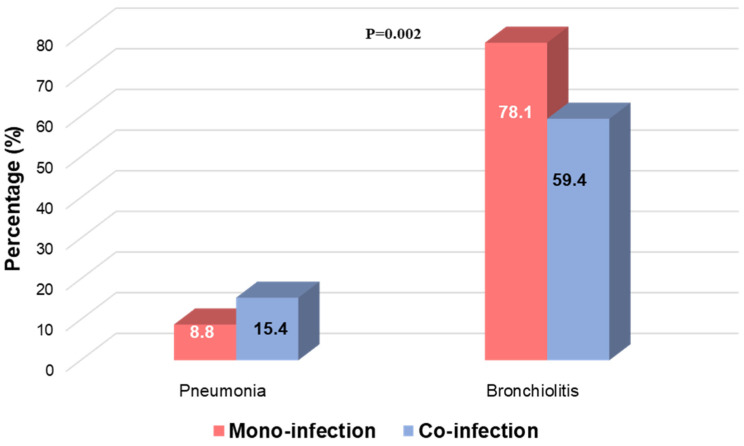
Distribution of bronchiolitis and pneumonia according to RSV mono-infection and RSV viral co-infection. Two-sided *p*-value was calculated by Fisher exact test.

Focusing on severe clinical manifestations, we observed that patients with a clinical diagnosis of pneumonia were more characterised by RSV viral co-infection (N = 48, 15.4%), whereas patients with bronchiolitis were mainly RSV mono-infected (N = 196, 78.1%) (*p*-value = 0.002) (Figure 3).

Based on these observations, univariate and multivariate logistic regression models were performed to investigate whether pneumonia was potentially associated with the type of RSV infection, RSV CT values and demographic characteristics (Table 3). As confounding factors, age, gender, sample type and RSV subtype were considered. The results showed that in our population, pneumonia was positively associated with increasing age (adjusted odds ratio, AOR [95% CI]: 1.48 [1.31–1.68], *p*-value < 0.001), higher RSV CT value (AOR [95% CI]: 1.07 [1.02–1.11], *p*-value = 0.006) and RSV co-infection compared to mono-infection (AOR: 1.97 [1.06–3.64], *p*-value = 0.031) (Table 3). On the other hand, the bronchiolitis-specific regression model showed a significant association between bronchiolitis and RSV mono-infection, younger age and lower RSV CT values.

These data were confirmed in a sub-analysis conducted on the population of children <1 year old. In particular, it was observed that bronchiolitis is more frequently associated with mono-infection. Additionally, a Spearman correlation analysis showed that age is positively correlated with CT values (r: 0.100, *p* = 0.044) and with the presence of co-infection (r: 0.256, *p* < 0.001), and negatively correlated with the length of hospital stay (r: −0.140, *p* = 0.004). This observation was further supported by a comparison between children under 1 year of age and those older than 1 year.

### 3.7. Correlations Between Respiratory Viruses

In addition, Spearman’s correlation test showed a correlation between co-infection of pneumonia patients and specific respiratory viruses. In particular, a positive correlation was observed in the case of pneumonia with AdV (r: 0.253, *p*-value = 0.038), HBoV (r: 0.320, *p*-value = 0.008), OC43 (r: 0.270, *p*-value = 0.026), Flu-A (r: 0.287, *p*-value = 0.018) and HRV (r: 0.462, *p*-value < 0.001). In bronchiolitis, only negative correlations with other viruses were mainly observed. Specifically, RSV was negatively correlated with AdV (r: −0.215, *p*-value < 0.001), NL63 (r: −0.122, *p*-value = 0.035), HeV (r: −0.176, *p*-value = 0.002), Flu-A (r: −0.121, *p*-value = 0.037), PIV-3 (r: −0.124, *p*-value = 0.032) and PIV-4 (r: −0.122, *p*-value = 0.035). This is in support of the fact that RSV in bronchiolitis is more likely to be alone.

## 4. Discussion

Respiratory syncytial virus is a major cause of respiratory infection in the paediatric population worldwide, often leading to severe outcomes such as hospitalisation and respiratory distress, particularly in infants and young children [16]. RSV presents as a heterogeneous disease with a wide spectrum of clinical manifestations influenced by several factors, such as age [17]. Although bronchiolitis in healthy infants accounts for the majority of hospitalisations for RSV infection, it is crucial to recognise that the disease can affect other age groups and can manifest itself with different clinical phenotypes [18]. In view of the widespread impact of RSV infections, it is crucial to understand the factors contributing to disease severity in order to improve clinical management and patient outcomes. This also encompasses the implementation of immunoprophylactic strategies, such as the nirsevimab use, to improve the management of RSV infections and determine the optimal conditions for these therapies to prevent complications in paediatric patients. Although these strategies are promising, further clinical evidence is needed to fully establish their efficacy in different paediatric populations. In particular, it is essential to identify the specific groups of children who would benefit most from these preventive measures. Therefore, this study investigated the relationship between RSV viral load, co-infection with other respiratory viruses and their impact on disease severity in a large cohort of paediatric patients followed at the Bambino Gesù Children’s Hospital in Rome.

A preliminary epidemiological analysis was conducted to assess the trend of RSV infections during the study period.

The RSV seasonal pattern observed during the study period was consistent with general expectations, showing higher incidence rates in the paediatric population during the colder months, a phenomenon influenced by several factors, including increased indoor activity and natural seasonality of respiratory viruses [15,19,20]. In particular, our data showed a shift from the predominance of RSV-B in late 2022 and early 2023 to the predominance of RSV-A in late 2023 and early 2024. The circulation of RSV subtypes is known to vary both geographically and seasonally, and even within a single season, with the dominant subtype varying from year to year, although both subtypes generally co-circulate during a given season [21,22].

In addition to the variation in the predominance of RSV subtypes, the present study showed that although RSV mono-infections are common among the paediatric patients studied, a significant percentage of them also have co-infections (55.1%) with other respiratory viruses, suggesting a complex interaction between the different circulating viruses. In our population, the majority of co-infections (62.7%) were due to human rhinoviruses. The latter are important pathogens of both upper and lower respiratory tract (particularly in patients with comorbidities), and co-infection with HRV can exacerbate inflammation by triggering rapid production of cytokines and chemokines that cause respiratory symptoms, especially in susceptible people [23]. In addition to HRV, this study found significant co-infections of RSV with human-bocavirus, human-enterovirus, adenovirus and the coronavirus OC43. These viruses are common in children with viral respiratory diseases and their co-presence with RSV has been associated with different clinical outcomes [24,25,26,27].

Previous studies have reported that viral co-infections, regardless of RSV involvement, are common in paediatric populations, but their impact on the clinical course is still debated, especially in the lower airways [28,29,30,31,32]. Most studies have focused on the impact of viral co-infections on bronchiolitis. Bermúdez-Barrezueta et al. showed that viral co-infections can accelerate the progression of acute bronchiolitis in hospitalised patients, prolong the duration of hospitalisation and increase C-reactive protein levels [33].

In our cohort, patients with RSV infection and bronchiolitis were predominantly characterised by RSV mono-infections (78.1%) with higher viral loads than those observed in other forms of lower respiratory tract infection. These findings were confirmed in a sub-analysis conducted in the group of children under one year of age, suggesting that even in the first year of life, as age increases, the likelihood of co-infection and higher Ct values increases, while the length of hospital stay decreases.

Overall, this suggests that, when unchallenged by other viruses, RSV may replicate more efficiently and exert a stronger cytopathic effect, particularly in younger children. This is consistent with the known pathogenesis of RSV, which primarily targets epithelial cells in the bronchiolar airways and alveolar regions [34]. The resulting cytopathology includes the abnormal proliferation of infected cells and the formation of multinucleated, polypoid epithelial masses that can obstruct the bronchiolar lumen [34]. In infants, the small calibre of the airways further increases the risk of severe obstruction and respiratory failure. The combination of direct viral damage and local inflammation leads to epithelial sloughing and airway occlusion mechanisms [23] that likely contribute to the more severe clinical presentation observed in RSV mono-infections within our study population.

In addition, Scagnolari et al. suggest that the lack of protective antibodies in infants with bronchiolitis favours viral replication, leading to a high viral load of RSV, a factor closely correlated with clinical severity and duration of hospitalisation [35].

A different picture was seen for pneumonia, which was positively associated with RSV co-infection, with a lower RSV viral load than in mono-infected bronchiolitis. In particular, viruses such as AdV, HBoV, OC43, Flu-A and HRV were positively associated with the development of pneumonia in our population. In this context, this observation emphasises that the major damage is not due to RSV alone, but to the presence and cooperation of other viruses that may act differently on respiratory tissue, particularly at the alveolar level (perhaps not in the bronchioles, see above). This finding adds important evidence to the literature, particularly in the paediatric setting, suggesting that co-infections, particularly with specific viruses such as AdV, HBoV, HRV, Flu-A, may exacerbate disease severity, leading to pneumonia and prolonged hospitalisation in RSV-infected patients. Furthermore, our results support the hypothesis that mixed infections in certain parts of the respiratory apparatus may promote more severe forms of disease, likely resulting from an intensified inflammatory response or impairment of the immune system’s ability to effectively control the primary virus. Babawale et al. reported that co-infections can lead to more complex and severe inflammatory responses and are associated with an increased risk of serious complications [28]. For example, in the adult population, the combination of influenza and RSV was found to be associated with significantly more severe outcomes than either influenza or RSV infection alone [36].

Another aspect analysed in this study is RSV viral load, the results of which provide valuable insights into the dynamics of RSV infection and its potential impact on disease severity. In this respect, the available data in the literature are limited in number and somewhat contradictory. Additionally, they only assess the difference in viral load between the two subtypes, without considering it as a potential factor that could influence clinical outcome. Indeed, Begley et al. observed a significantly higher viral load for RSV-B than for RSV-A, regardless of the sampling time between the two subtypes, and found a positive correlation between viral load and disease duration [37]. In contrast, in hospitalised infants, Rodriguez-Fernandez et al. found that RSV-A samples had higher viral loads than RSV-B samples [38].

Of particular note in this study is the analysis of the RSV viral load in relation to infections of the upper and lower respiratory tract. Our data reveal important differences in RSV viral load by site of infection and subtype, providing further evidence that viral load may be an indicator of disease severity in both mono- and co-infection scenarios. Far lower CT levels were observed in lower respiratory tract infections compared to upper respiratory tract infections for RSV-A and RSV-B, despite the specimens being from nasopharyngeal swabs (which primarily harvest the virus in the upper respiratory tract). This strongly supports the hypothesis of higher viral loads in the lower respiratory tract. The use of nasopharyngeal swabs in this study, which typically reflect upper respiratory viral loads, supports the hypothesis that RSV may replicate at higher levels in the lower respiratory tract during infection. Similarly, lower respiratory tract infections tend to have higher RSV viral loads, which may influence clinical outcomes, according to a study by El Saleeby et al. [34]. The authors found that higher viral loads were strongly predictive of longer hospital stay, development of respiratory failure on day 3, and were associated with an increased risk of needing intensive care [39].

The higher viral loads observed in RSV mono-infections in our study, indicated by significantly lower CT values, suggest that RSV replicates more efficiently when not competing with other pathogens. This enhanced replication likely leads to increased epithelial cell death and a stronger inflammatory response, resulting in greater airway damage. Such mechanisms may contribute to the increased severity and prolonged recovery commonly seen in infants with primary RSV infection [23]. Conversely, co-infections were associated with lower RSV viral loads, which may be explained by viral interference, where the presence of other viruses limits RSV replication. Alternatively, the immune response elicited by multiple pathogens during co-infections might modulate viral dominance, either suppressing or, in some cases, synergistically enhancing replication [28]. These findings underscore the complex interplay between RSV and other respiratory viruses and highlight the need for further research to clarify how these interactions influence disease severity and outcomes.

Observations on viral load, together with the results on pneumonia and bronchiolitis described above, further support the hypothesis that RSV alone, infecting and freely replicating in the bronchial lumen (without interference from other viruses) increases its cytopathic effect, inflammatory response and induces damage, whereas in co-infection the damage is probably induced by the action of different viruses present, and therefore does not require a high RSV viral load.

The viruses involved in the co-infections also play an important role in triggering virus-specific responses and thus the outcome; unfortunately, it was not possible in our context to determine the exact timing and order of infection of the viruses involved in co-infections. This is a potential limitation of the study. Another limitation is the lack of data on possible bacterial co-infections; this is a completely different area of the study, which requires a separate and dedicated focus. Similarly, the lack of data on the time between the onset of symptoms and the diagnosis or sample collection did not allow for a more detailed contextualisation of the findings. In addition, another limitation of our study is the lack of detailed data on the severity scale and ICU admission criteria for all patients. These clinical parameters were not easily retrievable across the entire study population, which may limit the detail of our analysis regarding disease severity and the need for intensive care. Lastly, there was no information on the patients’ vaccination status, such as influenza vaccination, as this can affect viral dynamics and kinetics, although the likelihood of children being vaccinated is very low, and the coverage of pregnant women with the influenza vaccine is also very scarce, so it is unlikely to be a factor, at least in this context.

Future prospective studies using comprehensive pathogen panels and focusing on the timing and progression of co-infections and on the dynamics of viral load at different stages of infection would further clarify the role of co-infection and viral load in the severity of RSV-related disease.

## 5. Conclusions

This study highlights the crucial role of RSV in paediatric respiratory infections, particularly in infants and young children. Co-infection with RSV and other respiratory pathogens was shown to be an important factor influencing disease type (upper vs lower respiratory infection and, in the latter case, bronchiolitis vs pneumonia), disease severity and viral load, which could be modulated by viral interference phenomena or immune responses. These findings highlight the importance of early and accurate diagnosis, accompanied by a personalised approach to the management of paediatric respiratory infections, with potential benefits for patient clinical outcomes. In addition, the study provides valuable insights into the pathogenesis of RSV infection and its complications, which could guide the use of these new immunoprophylactic measures. The use of multiplex tests, able to provide information about different pathogens present, as well as of the burden of the pathogen(s), makes it possible the identification of the infants most at risk of severe RSV infection, and will contribute to the optimisation of the indications for maternal vaccination and the administration of new immune-prophylaxis strategies aimed at substantially reducing the burden of disease.

## Figures and Tables

**Table 1:** Demographic characteristics of patients according to ARI.

	Overall	Upper ARI	Lower ARI	*p*-Value *
Total, N	637	88	549	
**Demographic characteristics**				
Male, N (%)	331 (52.0)	46 (52.3)	285 (51.9)	1.000
Age, months, median (IQR)	4.7 (1.8–20.9)	31.1 (10.5–58.6)	3.5 (1.7–16.2)	**<0.001**
Neonates, N (%)	53 (8.3)	1 (1.1)	52 (9.5)	**0.006**
Infant, N (%)	360 (56.5)	23 (26.1)	337 (61.4)	**<0.001**
Toddler, N (%)	75 (11.8)	16 (18.2)	59 (10.7)	0.051
Early Childhood, N (%)	117 (18.4)	37 (42.0)	80 (14.6)	**<0.001**
Middle Childhood, N (%)	21 (3.3)	6 (6.8)	15 (2.7)	0.056
Adolescents, N (%)	11 (1.7)	5 (5.7)	6 (1.1)	**0.011**
**Sample type**				
Nasopharyngeal Aspirate, N (%)	486 (76.3)	65 (73.9)	421 (76.7)	0.719
Nasopharyngeal Swab, N (%)	150 (23.5)	23 (26.1)	127 (23.1)	
Bronchoalveolar Lavage, N (%)	1 (0.2)	0 (0.0)	1 (0.2)	
**Clinical characteristics**				
Hospitalisation, N (%)	631 (99.1)	87 (98.9)	544 (99.1)	0.592
Length of hospitalisation Days, median (IQR)	5.9 (3.1–8.7)	5.0 (3.0–10.0)	5.9 (3.6–8.5)	0.536
Asthmatic bronchitis, N (%)	108 (17.0)		108 (19.7)	
Bronchiolitis, N (%)	373 (58.6)		373 (67.9)	
Pneumonia, N (%)	68 (10.7)		68 (12.4)	
**RSV genotype**				
RSV A, N (%)	312 (49.0)	48 (54.5)	264 (48.1)	0.301
RSV B, N (%)	334 (52.4)	40 (45.5)	294 (53.6)	0.169

* Two-sided *p*-values were calculated by Mann–Whitney test or Fisher exact test. Neonates: birth–28 days, Infant: 29 days–12 months, Toddler: 13 months–2 years, Early Childhood: 2.1–5 years, Middle Childhood: 6–11 years, Adolescence: 12–18 years. ARI: acute respiratory infection. IQR: interquartile range.

**Table 2:** RSV Cycle Threshold (CT) according to the type of infection and the type of respiratory tract involved.

RSV Cycle Threshold, Median (IQR)	Upper	Lower	*p*-Value *
**Mono-infection**	25.2 (19.6–28.4)	22.0 (18.9–25.7)	0.039
**Co-infection**	32.1 (24.3–38.3)	22.9 (19.7–27.4)	<0.001
***p*** -value *	0.002	0.037	

* Two-sided *p*-values were calculated by Mann–Whitney.

**Table 3:** Multivariate logistic regression analysis of factors associated with pneumonia.

Variable Associated Pneumonia	Univariable Analysis		Multivariable Analysis	
	OR (95% CI)	*p*-Value	AOR (95% CI)	*p*-Value
Gender (male vs. female)	0.92 (0.55–1.52)	0.736		0.907
Age (for 1 year increase)	1.52 (1.34–1.72)	<0.001	1.48 (1.31–1.68)	<0.001
Length of hospitalisation (Days, Median IQR)	1.03 (0.99–1.06)	0.131		0.295
Co-infection (vs. mono-infection)	1.90 (1.11–3.26)	0.020	1.97 (1.06–3.64)	0.031
*Type of sample* *:*				
Nasopharyngeal Aspirate	0.61 (0.31–1.20)	0.150		0.116
*RSV* *subtypes:*				
RSV A	1.09 (0.66–1.81)	0.736		0.520
RSV B	0.85 (0.51–1.41)	0.531		0.737
CT values (for 1 CT increase)	1.08 (1.02–1.14)	<0.001	1.07 (1.02–1.11)	0.006

Abbreviations: CI: confidence interval, CT: cycle threshold, OR: odds ratio, AOR: adjusted odds ratio. Reference categories: female for Gender; mono-infection for Co-infection; Nasopharyngeal Swab for Type of sample.

## Data Availability

Data are contained within the article (and its Supplementary Materials); further inquiries can be directed to the corresponding author.

## Supplementary Materials

The following supporting information can be downloaded at: https://www.mdpi.com/article/10.3390/v17091236/s1, Figure S1: Sample characteristics and inclusion criteria of the analysis; Figure S2: Temporal distribution of RSV in in the paediatric population from January 2022 to April 2024; Figure S3: Distribution of viruses involved in co-infections with RSV in the overall population (panel A) and according to upper and lower respiratory tract infections (panel B); Table S1: Seasonal patterns of RSV coinfections with other respiratory viruses.

## Abbreviations

The following abbreviations are used in this manuscript:

AdV	Adenovirus
AOR	Adjusted Odds Ratio
ARI	Acute Respiratory Infections
CI	Confidence Interval
CT	Cycle Threshold
Flu	Influenza
HBoV	Human Bocavirus
HeV	Human Enterovirus
HMPV	Human Metapneumovirus
HRV	Human Rhinovirus
ICU	Intensive Care Unit
IQR	Interquartile-Range
LTRI	Lower Respiratory Tract Infections
NCoV	Human Coronavirus
OR	Odds Ratio
PIV	Parainfluenza Viruses
RSV	Respiratory Syncytial Virus
SARS-CoV-2	Severe Acute Respiratory Syndrome-COronaVirus 2
URTI	Upper Respiratory Tract Infection
