# Supplementary material for: Viral Burden of Respiratory Syncytial Virus and Viral Coinfections as Factors Regulating Paediatric Disease Severity
Source: Viruses. 2025 Sep 11;17(9):1236. doi: 10.3390/v17091236 (PMC12474089; doi:10.3390/v17091236)
Supplement: Supplementary file 1 [file viruses-17-01236-s001.zip › SupplemTable1_new.pdf]

Supplementary Table S1. Seasonal patterns of RSV coinfections with other respiratory viruses.

		AdV (N=54)	*HBoV (N=70)*	*229E (N=5)*	*NL63 (N=3)*	*OC43 (N=42)*	*HEV (N=55)*	*Flu A (N=21)*	*Flu B (N=1)*	*MPV (N=20)*	*PIV1 (N=7)*	*PIV2 (N=5)*	*PIV3 (N=17)*	*PIV4 (N=4)*	*HRV A/B/C (N=220)*	*SARS-CoV-2 (N=22)*
2022	Jan-March	9.1	27.3	9.1	0	9.1	0	0	0	9.1	0	0	18.2	0	36.4	45.5
	Jul-Sept	0	0	0	0	0	0	0	0	0	0	0	50	0	50	50
	Oct-Dec	16.3	17.3	1	0	12.2	12.2	9.2	1	6.1	3.1	0	4.1	1	60.2	9.2
2023	Jan-March	21.3	14.9	4.3	2.1	19.1	12.8	8.5	0	17	2.1	2.1	4.3	2.1	48.9	4.3
	Apr-Jun	50	25	0	25	0	25	0	0	50	25	0	25	0	25	0
	Oct-Dec	12.1	13.8	0	1.7	6.9	13.8	1.7	0	0	0	1.7	5.2	1.7	72.4	3.4
2024	Jan-March	14	25.6	0.8	0	12.4	20.9	5.4	0	2.3	1.6	2.3	2.3	0.8	69	2.3
	Apr-Jun	0	50	0	0	0	50	0	0	0	0	0	50	0	50	0

Values are expressed as a percentage. The prevalence of pathogens was calculated based on the total number of coinfections detected in each trimester. ADV: Adenovirus, HBoV: Human bocavirus, HCoV-229E: Human coronavirus 229E, HCoV-OC43: Human coronavirus OC43, HeV: Human enterovirus, FluA: Influenza A virus, FluB: Influenza B virus; MPV: Human metapneumovirus, PIV1: Human parainfluenza virus 1, PIV2: Human parainfluenza virus 2, PIV3: Human parainfluenza virus 3, PIV4: Human parainfluenza virus 4, HRV: Human rhinovirus, RSVA: Respiratory syncytial virus A, RSVB: Respiratory syncytial virus B, SARS-CoV-2: Severe acute respiratory syndrome coronavirus.
