# Supplementary figures and images for: Viral Burden of Respiratory Syncytial Virus and Viral Coinfections as Factors Regulating Paediatric Disease Severity
Source: Viruses. 2025 Sep 11;17(9):1236. doi: 10.3390/v17091236 (PMC12474089; doi:10.3390/v17091236)

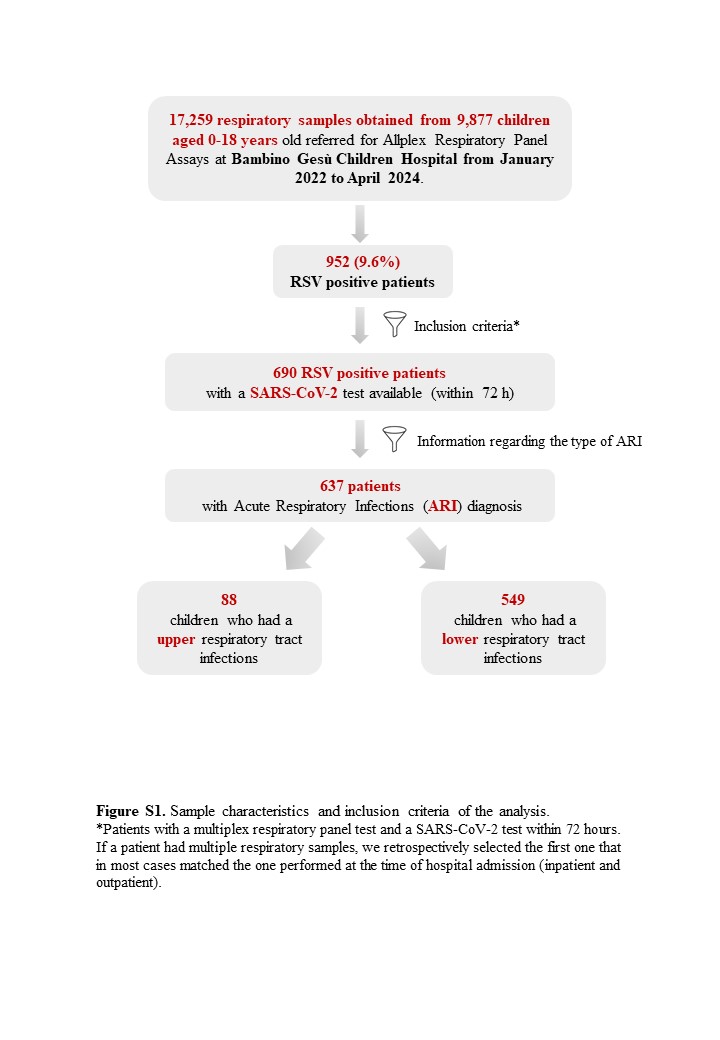

Supplement: Supplementary file 1 [file viruses-17-01236-s001.zip › Figure S1.jpg]

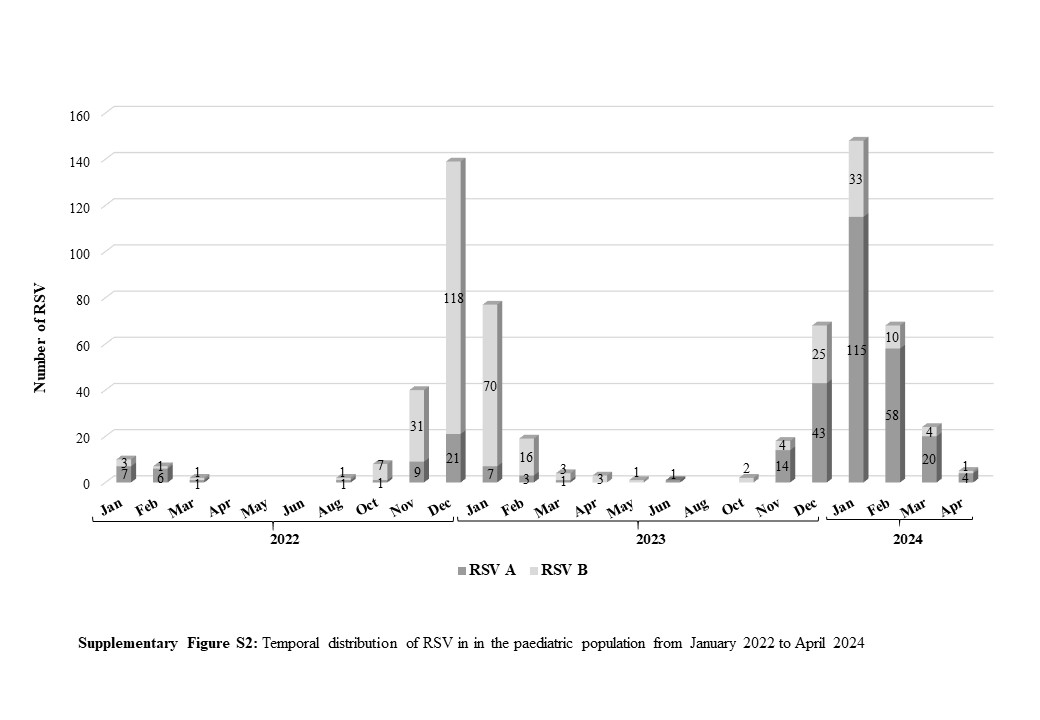

Supplement: Supplementary file 1 [file viruses-17-01236-s001.zip › FigureS2.jpg]

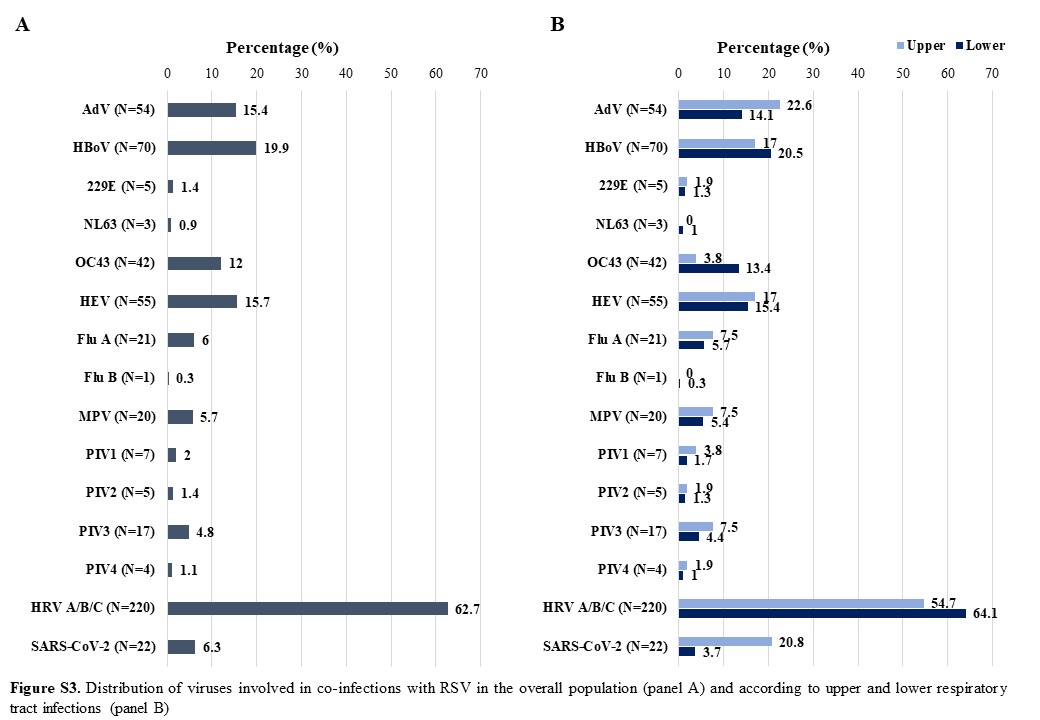

Supplement: Supplementary file 1 [file viruses-17-01236-s001.zip › FigureS3_rev.jpg]
